# Tuberculous Infection through the Alimentary Canal

**Published:** 1895-07

**Authors:** Sheridan Delapine

**Affiliations:** Professor of Pathology, Owens College


### SELECTIONS.

### Tuberculous Infection Through the Alimentary Canal.

When a truth, whether scientific or other, goes against the interests of a large and powerful class of the community, it is generally ignored or resisted till a more powerful class enforces its recognition. For the last thirty years farmers and butchers have been roughly disturbed by mere scientists, medical observers, and veterinary surgeons, who have come to the conclusion that it is not safe for man or beast to feed on tuberculous products.

At first the observations of Villemin, Chauveau, Gerlach, Günther and Harms, Toussaint, Peuch, Bollinger, Jöhne, etc., though conclusive enough, were not accepted as quite convincing by a number of authorities, because there was no easy criterion by which tuberculous lesions could be recognized.

Even after Koch's demonstration of the presence of a tubercle bacillus, not only in human tuberculous lesions, but also in those of the lower animals, many who considered themselves experts tried to evade the logical consequences of this discovery.

At the present time opinions are still divided, and thereby the work of the medical officer of health is rendered so difficult that he may at times be hindered from serving the public interests which he has to protect.

The evidence which has been adduced to prove that the flesh of tuberculous animals was not capable of causing tuberculosis, though very interesting, cannot shake in the least the observations on which the opposite view is based. It is with the object of showing the bearings of the arguments brought forward by both sides that I will now attempt to give a short account of the present state of this question.

It will be taken for granted that the infectiousness of tuberculosis has been fully demonstrated by the work of Villemin, of Koch, and of their followers. Tuberculosis of the lower animals is due to the same micro-organism as in man, and it can be communicated from man to the lower animals and from the lower animals to man.

This statement is not affected by the fact that there are certain differences in the morphology of the bacillus and in the character and mode of extension of the lesions in the two cases.

We need not either discuss the question of heredity as a causal factor in tuberculosis. It can be safely admitted that an exceedingly small number of children are affected at birth, and the same may equally truly be said of the young of the lower animals. The few cases of fatal tuberculosis recorded by Hiller, Shleuss and Grothaus, Jöhne, Landouzy and Martin, etc., though they show that it is impossible for a fœtus to be infected in utero, are of little importance when compared with the statistics given by Boltz, for instance, in his "Inaugural Dissertation." Out of 2576 children whose bodies were submitted to a post-mortem examination in Kiel, between 1873 and 1889, there were 424 cases of tuberculosis.

Woodhead has given, on a smaller scale, results equally significant of 127 cases of tuberculosis.

Such statistics, and others not mentioned here, indicate clearly that tuberculosis usually begins after birth, and that it is a rare disease in the early months of life. There is ample evidence to prove that among the channels of infection the air-passages are most important; a large number of children must, however, become infected through their alimentary canal.

In a series of over one hundred experiments made by myself I have found that tuberculosis of the mesenteric glands occurs very late, if at all, in guinea-pigs infected through other channels than the alimentary canal or the peritoneal cavity, and when the mesenteric glands are affected in such cases all the other organs are in a state of advanced tuberculosis; but where the seat of inoculation is in the abdominal cavity, the mesenteric glands are, on the contrary, the first organs affected. In fact, it is generally possible, by carefully dissecting out the lymphatic ganglia, to find the part through which the animal has been infected.

To give only a practical instance of the results obtained it will be enough to mention here that I have found a single prick of the tongue, of the lips, or of the fauces with a needle loaded with tubercle bacilli to be followed by marked tuberculosis of the cervical ganglia, the lesions in such a case resembling closely those observed in the neck of scrofulous children.

In the presence of such observations it is only right to assume that when the mesenteric glands are the only organs affected with tuberculosis, or when they are much more affected than other organs, the channel of infection must have been the alimentary canal.

Judging from the statistics recorded above it is evident that this occurrence is far from infrequent in children. In corroboration of this fact we have seen that children begin to be liable to tuberculosis after they had reached the age of six months (*i. e.*, after most of them have begun to feed on cow's milk, or foods other than the maternal milk).

There is, however, more than circumstantial evidence to show the danger of tuberculous food. Chauveau, Gerlach, Harmz and Günther, Jöhne, Nocard, Peuch, Viseur, etc., have shown that tuberculous organs and flesh when given to various kinds of animals were capable of causing tuberculosis beginning in the abdomen. Gerlach, Klebs, Jöhne, Bollinger, Baumgarten, Peuch, Bang, etc., have proved the existence of the same danger in connection with the milk from tuberculous cows.

Nocard has pointed out that when cats are fed with muscle free from tuberculous glands, and not accidentally contaminated with tuberculous products coming from other organs, tuberculosis is never obtained. This is true even when young kittens are used, though these animals are easily rendered tuberculous by the ingestion of small quantities of tuberculous lungs, liver, and lymphatic ganglia.

In support of Nocard's contentions we have the experiments of Peuch, Galtier, and Perroncito, showing that muscular tissue taken from tuberculous animals, but free from tubercular lesions, is practically incapable of producing tuberculosis by ingestion.

There is no doubt as to the danger of eating tuberculous food, but there is a divergence of opinion among authorities as to the parts of an animal which are infectious.

Various attempts have been made to prove that though this is not always true, the blood can at times contain enough tubercle bacilli to render any part of the body infectious. This, however, has been shown by Bang, Nocard, and McFadyean to be an extremely rare occurrence.

The only thing which has been clearly proved is that whenever an organ shows distinct tuberculous lesions this organ is capable of transmitting tuberculosis by inoculation or ingestion.

It is, therefore, interesting to find what is the prevalence of tuberculosis in the various organs of bovidæ which, of all the animals used for food, are most liable to tuberculosis. According to Nocard, the organs of oxen and cows are affected in the following order of frequency: Lungs and pleura, 40 per cent.; lungs alone, 20 to 25 per cent.; pleura and peritoneum alone,

15 to 20 per cent.; lymphatic glands, genital organs, mammæ, bones, articulations, 15 to 25 per cent.

Nocard insists upon the fact that it is not very unusual to find lymphatic ganglia affected without there being any trace of tuberculosis in the viscera through which, he assumes, the tubercle bacilli have penetrated into the organism. This is not only true of the thoracic and abdominal ganglia, but also of those which are outside the cavities, notably the cervical and pharyngeal ganglia.

A slight knowledge of the distribution of lymphatic ganglia in the body will make it evident that there is hardly any joint of meat, as cut by the butcher, which does not contain one or more lymphatic glands. These ganglia have often been found to be diseased not only in the neighborhood of the great serous cavities, but also in the extremities. This may even be the case without the meat showing any evidence of the existence of a wasting disease; in fact, tuberculous glands have been found in meat apparently of prime quality. Such an invasion of the peripheral lymphatics is, of course, generally most extensive in advanced cases in which wasting has taken place; the appearance of the meat in such instances easily betrays its unsoundness.

If this invasion of the lymphatics is taken in consideration, the careful experiments by which several observers had tried to prove that the flesh of tuberculous animals is not infectious, by carefully separating the muscular from the surrounding tissues, are not of much practical value, notwithstanding their great scientific interest.

But it is not only through the presence of tuberculous glands in their midst that meat from tuberculous animals is rendered dangerous. Butchers, when they dress a carcass, do not, like the careful experimenter, sterilize their fingers and knives at each step of the dissection; they do not sterilize the walls, tables, hooks, etc., which they use after these have come in contact with tuberculous carcasses or offal. On the contrary, the meat constantly runs the risk of being contaminated with discharges or products coming from the tuberculous organs which no doubt are condemned and destroyed, but leave traces behind them.

What is, therefore, the practical use in experiments on the infectiousness of flesh from tuberculous animals, of muscles or blood, removed or collected under conditions which cannot be observed in the abattoir?

It is fortunate for us that it is only when the tubercle bacilli are pretty abundant that they are capable of causing disease in a healthy adult person. But in weak or young individuals the danger is much greater, and there is always a possibility that some bone or hard particle infected with tuberculous matter may wound the mucous membrane of the mouth, fauces, or some other part of the alimentary canal, and bring about tuberculosis just as in my experiments with the tuberculous needle.

It is not easy to prove the actuality of the danger by quoting cases in which tuberculosis has been undoubtedly the result of the ingestion of tuberculous meat.

The statistics to prove the frequency of abdominal tuberculosis in children appeal only to those who have made a large number of experiments and observations.

Even in those cases in which meat might be suspected to be the cause of tuberculosis it would be difficult to trace clearly the piece of meat which should be incriminated, and afterward to establish clearly its history; and if this could be done it is evident that the carcass from which the joint has been obtained would have long been beyond the reach of scientific investigation when tuberculosis had made sufficient progress in the victim to be evident.

With regard to infection by milk, however, it is sometimes possible to find a case showing clearly that infection has been due to the milk.

Demme mentions four cases occurring in children of healthy parents. Those children were clearly proved to have been fed on uncooked milk from tuberculous cows.

Ollivier and Boutet, quoted by Arloing, record an outbreak of tuberculosis in a boarding-school. Cows were kept in that establishment. One of the cows supplying milk during a certain period was tuberculous, and during that time six of the children became tuberculous.

Stang gives details of another case. A boy, aged five years, born of healthy parents, died from advanced tuberculosis of the mesenteric ganglia and miliary tuberculosis of the lungs. It was ascertained that he had been in the habit of drinking warm uncooked milk from a cow suffering from tuberculosis, as was proved by examination of the viscera after the animal had been slaughtered.

A still more telling instance is recorded by Dr. Gosse, of Geneva. His daughter was, at the age of sixteen years, a strong and healthy girl, and there was no trace of tuberculosis in the family. About ten months before her death she began to manifest signs of some mysterious illness, which caused gradual wasting, and ultimately the patient died at the age of seventeen years. At the autopsy it was found that the poor girl had suffered from intestinal and mesenteric tuberculosis. It was then remembered that she used, when spending the Sundays on a small estate where cows were kept, to drink warm milk almost straight from the cows. These cows were in consequence of this examined; four out of five were found tuberculous, and of these two had tuberculous udders.

Such instances of infection, though few, are sufficiently clear to establish the risks attending the use of tuberculous food. It may be argued that meat is usually cooked before being eaten, and that the virulence of the tubercle bacillus is in this way destroyed; but it must be remembered that a large proportion of the meat used for food is either roasted or grilled, and that the internal parts of the chops, beefsteaks, or roasted joints are seldom sufficiently cooked to kill bacilli.

Chauveau and Arloing have shown that unless a tuberculous piece of meat has been kept all through at a temperature of 70° C. for about half an hour it is still capable to infect animals. Now whenever the inside of a joint is still red it can safely be said that the temperature of the red parts has not even reached 70° C.

Bang has shown that after milk has been kept at a temperature of 85° C. for about five minutes any tubercle bacilli which it might contain are killed. To obtain such a temperature in the centre of an ordinary joint much more prolonged cooking than is generally desired should be allowed.

Such are the main facts and arguments which may be relied upon to prove the danger connected with the consumption of tuberculous meat. Many more observations and experiments might have been quoted, but it has been thought that they could hardly be more convincing than those alluded to in the text. Only few bibliographical references are given, because most of the information wanted will be easily found in works such as those of Arloing and Nocard, and also in papers by Woodhead and McFadyean.

It is hoped that the facts brought together in this short *exposé* will be enough to convince the reader of the seriousness of the matter at issue, and of the real risk which is incurred if tuberculous meat is allowed to pass as fit for human food by meat inspectors.

(The report of the Royal Commission on Tuberculosis not being available yet to the public, I regret not to be able to take advantage of it in support of my contention, but it may be said in a general way to establish the views here advanced.)—SHERIDAN DELAPINE, *Professor of Pathology, Owens College, in The Veterinary Record*, June 1, 1895.

